# Circulating biomarkers in subjects with progressive pulmonary fibrosis: data from the INBUILD trial

**DOI:** 10.1183/23120541.00158-2025

**Published:** 2026-02-23

**Authors:** Toby M. Maher, R. Gisli Jenkins, Francesco Bonella, Shervin Assassi, Claudia Diefenbach, Carina Ittrich, Klaus B. Rohr, Martin Kolb

**Affiliations:** 1 National Heart and Lung Institute, Imperial College London, London, UK; 2 Keck School of Medicine, University of Southern California, Los Angeles, CA, USA; 3 National Heart and Lung Institute, Imperial College London, London, UK; 4 Center for Interstitial and Rare Lung Diseases, Ruhrlandklinik University Hospital, Duisburg-Essen University, Essen, Germany; 5 Division of Rheumatology, University of Texas McGovern Medical School, Houston, TX, USA; 6 Boehringer Ingelheim Pharma GmbH & Co. KG, Biberach an der Riss, Germany; 7 Boehringer Ingelheim International GmbH, Ingelheim am Rhein, Germany; 8 McMaster University and St Joseph's Healthcare, Hamilton, ON, Canada

## Abstract

**Background:**

We investigated the prognostic potential of circulating biomarkers at baseline and the effects of nintedanib on changes in these biomarkers in subjects with progressive pulmonary fibrosis (PPF).

**Methods:**

In the INBUILD trial, subjects with PPF received nintedanib (n=332) or placebo (n=331). Associations between biomarker levels at baseline and the rate of forced vital capacity (FVC) decline (mL·year^−1^) over 52 weeks, time to interstitial lung disease (ILD) progression (absolute decline in FVC % predicted ≥10%) or death over 52 weeks, time to first acute exacerbation or death over the whole trial, and time to death over the whole trial were assessed in the placebo group. Changes in adjusted mean levels of biomarkers in the nintedanib and placebo groups were assessed using linear mixed models for repeated measures. Biomarker data were log_2_ transformed prior to analysis. Analyses were corrected for multiplicity.

**Results:**

Baseline level of s-ICAM was significantly associated with rate of FVC decline and time to ILD progression or death over 52 weeks in the placebo group. No biomarker was significantly associated with time to first acute exacerbation or death or time to death. Decreases in Krebs von den Lungen-6 (KL-6), surfactant protein D (SP-D), CA-125 and CA19-9 were observed in subjects who received nintedanib *versus* placebo over 52 weeks. The largest decrease was in CA-125. In a mediation analysis, 16.4% of the effect of nintedanib on change in FVC at week 52 was attributed to the treatment-related decrease in CA-125 at week 12.

**Conclusions:**

In subjects with PPF, nintedanib reduced circulating CA-125 and, to a lesser extent, other markers of epithelial dysfunction.

## Shareable abstract

**In a randomised controlled trial in subjects with progressive pulmonary fibrosis, nintedanib reduced circulating levels of markers of epithelial dysfunction, particularly CA-125, *versus* placebo** https://bit.ly/45ACTTC

## Introduction

The term progressive pulmonary fibrosis (PPF) is generally used to describe progressive lung fibrosis in a patient with an interstitial lung disease (ILD) other than idiopathic pulmonary fibrosis (IPF) [1]. Although different criteria are used to define PPF, based on varying parameters of decline in lung function, worsening of symptoms and worsening of radiological abnormalities [1–4], progression of pulmonary fibrosis is invariably associated with poor outcomes [5, 6].

The development of lung fibrosis involves interplay among inflammatory, immune and fibrotic mechanisms. The current paradigm proposes that repeated epithelial injury results in the release of pro-inflammatory and pro-fibrotic mediators, the differentiation of fibroblasts into myofibroblasts and excess deposition of extracellular matrix (ECM), which further activates fibroblasts, ultimately leading to a self-sustaining process of fibrosis [7]. Nintedanib, an intracellular inhibitor of tyrosine kinases, shows both anti-inflammatory and antifibrotic effects in nonclinical studies [8]. In placebo-controlled clinical trials, nintedanib slowed the rate of decline in forced vital capacity (FVC) in subjects with IPF [9], ILD associated with systemic sclerosis [10] and PPF [2].

The clinical course of PPF is variable [11, 12]. Thus, the identification of circulating biomarkers prognostic of short-term outcomes, or indicative of response to nintedanib in an individual with PPF, would be of clinical value. In this analysis, we used data from the INBUILD trial to evaluate the prognostic potential of circulating biomarkers and the effects of nintedanib on these biomarkers in subjects with PPF.

## Methods

### Trial design

The design of the INBUILD trial (NCT02999178) has been published [2]. In summary, subjects had an ILD other than IPF, an extent of fibrosis on high-resolution computed tomography (HRCT) >10% and FVC ≥45% predicted. Subjects met ≥1 of the following criteria for ILD progression within the prior 24 months despite management in clinical practice: relative decline in FVC % predicted ≥10%; relative decline in FVC % predicted ≥5–<10% and increased extent of fibrosis on HRCT and/or worsened respiratory symptoms; worsened respiratory symptoms and increased extent of fibrosis on HRCT [2]. Subjects were randomised to receive nintedanib or placebo, stratified by fibrotic pattern on HRCT (usual interstitial pneumonia (UIP)-like fibrotic pattern or other fibrotic patterns).

The INBUILD trial was carried out in compliance with the principles of the Declaration of Helsinki and the Harmonised Tripartite Guideline for Good Clinical Practice from the International Conference on Harmonisation and was approved by local authorities. All subjects provided written informed consent before trial entry.

### Blood sample collection and biomarker analysis

Samples for biomarker analysis were collected at baseline and at weeks 12, 24, 36 and 52. Serum samples were prepared using anticoagulant-free, gel-containing serum separation tubes, which were left to clot for ∼1 h at room temperature. Plasma samples were prepared using K_2_ EDTA plasma tubes. Serum and plasma were separated by centrifugation and aliquoted prior to freezing. Krebs von den Lungen-6 (KL-6), surfactant protein D (SP-D), CA-125 and CA19-9 were assessed as biomarkers of epithelial dysfunction. C-reactive protein (CRP), interleukin-8 (IL-8) and soluble intercellular adhesion molecule (s-ICAM) were assessed as biomarkers of inflammation. Biglycan degraded by MMP (BGM), collagen 1 degraded by MMP-2/9/13 (C1M), collagen 3 degraded by MMP-9 (C3M), collagen 5 degraded by MMP-2/9 (C5M), collagen 6 degraded by MMP-2/9 (C6M), neutrophil-specific elastin fragments (EL-NE), MMP-7, CRPM, N-terminal propeptide of type III collagen (pro-C3) and N-terminal propeptide of type VI collagen (pro-C6) were assessed as biomarkers of ECM turnover. Commercially available ELISA methods were used to measure plasma concentrations of KL-6 and SP-D. The Sanko Junyaku Co., Ltd./EIDIA Co., Ltd test kit was used for KL-6, with the assay performed according to the manufacturer's protocol. The human SP-D ELISA test kit from Biovendor was used for SP-D. Serum concentrations of CA-125 and plasma concentrations of s-ICAM and CA19-9 were measured using an electrochemiluminescence immunoassay. Concentrations of CA-125 were assessed using the CA-125 antigen Beckman DxI 800 assay (a two-site immunoenzymatic assay). Concentrations of s-ICAM were assessed using the Vascular Injury Panel 2 (human) K15198 test kit from Meso Scale Discovery. CRP concentrations were measured *via* immunonephelometry using the Siemens BNII Nephelometer. IL-8 concentrations were measured using the Quantikine Human CXCL8/IL-8 enzyme immunoassay. Serum concentrations of the biomarkers of ECM turnover were measured using ELISA methods.

### Statistical analyses

Spearman's correlation coefficients (rho) between FVC (mL) and concentrations of each biomarker at baseline were calculated in all patients (nintedanib and placebo). Associations between baseline biomarker levels and the following end-points were assessed in the placebo group: rate of decline in FVC (mL·year^−1^) over 52 weeks; time to ILD progression (absolute decline in FVC % predicted ≥10%) or death over 52 weeks; time to first acute exacerbation or death over the whole trial; and time to death over the whole trial. Biomarker data were not normally distributed and were log_2_ transformed prior to analysis. The rate of decline in FVC (mL·year^−1^) over 52 weeks was analysed using random coefficient regression. Estimates represented the rate of decline in FVC (mL·year^−1^) over 52 weeks associated with a difference of one in the log_2_ transformed level of the biomarker at baseline. Time to event end-points were analysed using Cox regression models. Hazard ratios (HR) represented the risk of an event associated with a difference of one in the log_2_ transformed level of the biomarker at baseline. Analyses were adjusted for baseline FVC % predicted (or FVC in mL for the rate of decline in FVC in mL·year^−1^), diffusing capacity of the lung for carbon monoxide % predicted, age, sex, HRCT pattern (UIP-like pattern or other fibrotic patterns), inclusion criteria for ILD progression and batch (for C3M, CRPM, pro-C3 and pro-C6). p-values were corrected for multiple comparisons using the Benjamini–Hochberg method [13] to control the false discovery rate (FDR) at 5%.

Changes from baseline in adjusted mean levels of biomarkers at each visit were assessed in the overall population and in subgroups by HRCT pattern using linear mixed models for repeated measures. Models included fixed categorical effects of treatment at each visit, sex, race, ILD subtype, inclusion criterion for ILD progression, batch (for C3M, CRPM, pro-C3 and pro-C6), and fixed continuous effects of the baseline value of the biomarker at each visit and age. Within−patient errors were modelled by an unstructured variance−covariance matrix. Biomarker data were log_10_ transformed before analysis.

Mediation analyses assessed the extent to which the effect of nintedanib on the change in a biomarker at week 12 was related to the effect of nintedanib on change in FVC at week 52. Biomarkers that demonstrated an indirect relationship with FVC with p<0.1 (*i.e.*, a notable relationship between change in the biomarker and change in FVC), a negative estimated slope of the regression line with p<0.05 (*i.e.*, a negative linear relationship between change in the biomarker and change in FVC) and a decrease with nintedanib *versus* placebo at week 12 with p<0.05 were included in mediation analyses. Correlations (rho) between changes in these biomarkers at weeks 12, 24, 36 and 52 and the rate of decline in FVC over 52 weeks were calculated.

## Results

### Subjects

A total of 663 subjects received trial medication (332 nintedanib, 331 placebo). Their baseline characteristics have been published [2]. The majority of subjects were male (53.7%) and white (73.6%). Mean±sd age was 65.8±9.8 years and mean±sd FVC was 69.0±15.6% predicted; 412 subjects (62.1%) had a UIP-like pattern on HRCT. The baseline levels of each biomarker are shown in table 1.

**TABLE 1:** Baseline values of biomarkers in the INBUILD trial

	Overall population		UIP-like fibrotic pattern on HRCT		Other fibrotic patterns on HRCT	
	Nintedanib	Placebo	Nintedanib	Placebo	Nintedanib	Placebo
**KL-6 U·mL^−1^**	1033±1016	1035±878	943±843	894±756	1182±1243	1274±1012
**SP-D ng·mL^−1^**	772±543	732±528	715±436	700±501	867±677	787±569
**CA-125 U·mL^−1^**	19.2±16.9	21.8±20.6	19.1±14.1	21.0±20.6	19.5±20.8	23.3±20.6
**CA19-9 U·mL^−1^**	48.7±82.0	47.6±83.4	53.4±89.3	45.3±73.1	40.4±66.8	51.4±98.2
**CRP mg·L^−1^**	6.8±12.9	6.1±13.1	7.7±15.4	6.7±15.8	5.3±6.6	5.1±6.1
**IL-8 ng·L^−1^**	29.8±36.6	33.5±108.1	31.1±38.7	36.5±132.2	27.6±32.9	28.4±42.5
**s-ICAM ng·mL^−1^**	714±230	710±242	707±213	681±205	727±257	758±289
**BGM ng·mL^−1^**	15.1±8.8	16.2±10.0	14.1±8.5	16.2±10.3	16.6±9.2	16.3±9.6
**C1M ng·mL^−1^**	48.1±37.0	49.5±39.5	47.8±36.1	49.0±41.8	48.6±38.5	50.4±35.6
**C3M ng·mL^−1^**	14.9±5.1	14.9±4.5	15.5±5.8	15.0±4.9	13.8±3.5	14.9±3.7
**C5M ng·mL^−1^**	8.1±4.2	8.1±3.9	8.2±4.3	8.5±4.2	7.8±4.2	7.4±3.3
**C6M ng·mL^−1^**	30.9±17.9	29.5±12.7	31.8±19.8	29.7±13.4	29.5±14.0	29.0±11.6
**EL-NE ng·mL^−1^**	6.7±6.6	7.5±6.9	6.2±6.2	7.5±7.2	7.6±7.3	7.4±6.5
**MMP-7 µgEq·L^−1^**	10.9±5.7	10.9±5.5	10.9±5.2	10.8±5.0	10.9±6.5	11.2±6.2
**CRPM ng·mL^−1^**	11.0±3.5	11.5±4.1	11.0±3.6	11.3±3.9	10.9±3.4	11.7±4.5
**Pro-C3 ng·mL^−1^**	13.4±6.0	14.1±6.5	13.7±6.3	14.6±7.2	13.0±5.4	13.3±5.1
**Pro-C6 ng·mL^−1^**	9.7±3.6	9.7±4.0	9.7±3.8	9.6±4.1	9.6±3.2	9.8±3.9

Data are presented as mean±sd unadjusted values. Not all subjects provided data on all biomarkers. n=284 to n=321 for nintedanib and n=287 to n=313 for placebo in the overall population. n=181 to n=201 for nintedanib and n=178 to n=197 for placebo among subjects with UIP-like fibrotic pattern on HRCT. n=103 to n=120 for nintedanib and n=109 to n=116 for placebo among subjects with other fibrotic patterns on HRCT. UIP: usual interstitial pneumonia; HRCT: high-resolution computed tomography; KL-6: Krebs von den Lungen-6; SP-D: surfactant protein D; CRP: C-reactive protein; IL-8: interleukin-8; s-ICAM: soluble intercellular adhesion molecule; BGM: biglycan degraded by MMP; C1M: collagen 1 degraded by MMP-2/9/13; C3M: collagen 3 degraded by MMP-9; C5M: collagen 5 degraded by MMP-2/9; C6M: collagen 6 degraded by MMP-2/9; EL-NE: neutrophil-specific elastin fragments; pro-C3: N-terminal propeptide of type III collagen; pro-C6: N-terminal propeptide of type VI collagen.

### Prognostic value of circulating biomarkers in the placebo group

**FIGURE 1:**
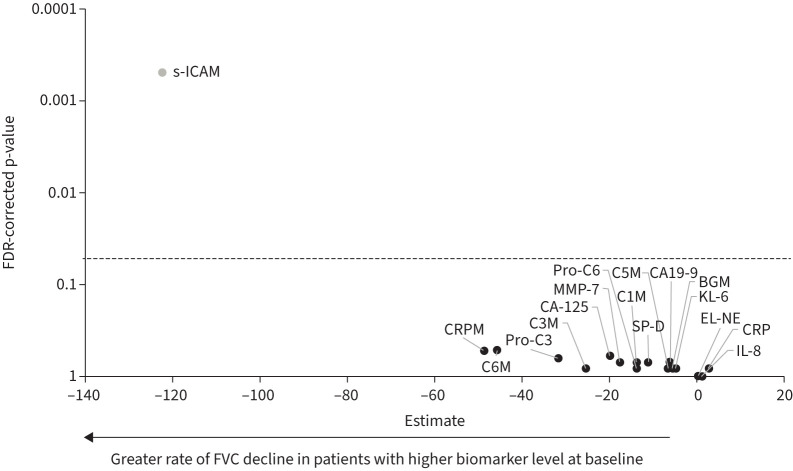
Associations between baseline biomarker levels and rate of decline in forced vital capacity (FVC) in the INBUILD trial. Estimates represent the rate of decline in FVC (mL·year^−1^) over 52 weeks associated with difference of one in the log_2_ transformed level of the biomarker at baseline. Biomarker shown as a grey circle had a significant association between the baseline value and the outcome (FDR-corrected p<0.05). FDR: false discovery rate; KL-6: Krebs von den Lungen-6; SP-D: surfactant protein D; CRP: C-reactive protein; IL-8: interleukin-8; s-ICAM: soluble intercellular adhesion molecule; BGM: biglycan degraded by MMP; C1M: collagen 1 degraded by MMP-2/9/13; C3M: collagen 3 degraded by MMP-9; C5M: collagen 5 degraded by MMP-2/9; C6M: collagen 6 degraded by MMP-2/9; EL-NE: neutrophil-specific elastin fragments; pro-C3: N-terminal propeptide of type III collagen; pro-C6: N-terminal propeptide of type VI collagen.

**FIGURE 2:**
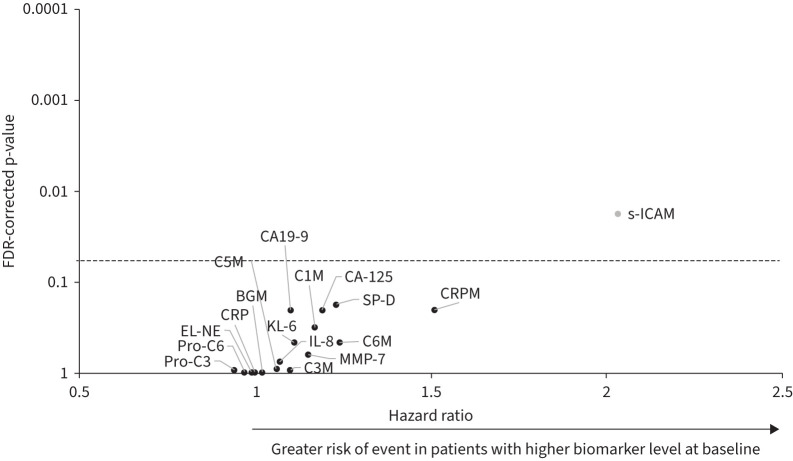
Associations between baseline biomarker levels and time to ILD progression or death over 52 weeks in the INBUILD trial. Hazard ratios represent the risk of an event associated with a difference of one in the log_2_ transformed level of the biomarker at baseline. Biomarker shown as a grey circle had a significant association between the baseline value and the outcome (FDR-corrected p<0.05). FDR: false discovery rate; KL-6: Krebs von den Lungen-6; SP-D: surfactant protein D; CRP: C-reactive protein; IL-8: interleukin-8; s-ICAM: soluble intercellular adhesion molecule; BGM: biglycan degraded by MMP; C1M: collagen 1 degraded by MMP-2/9/13; C3M: collagen 3 degraded by MMP-9; C5M: collagen 5 degraded by MMP-2/9; C6M: collagen 6 degraded by MMP-2/9; EL-NE: neutrophil-specific elastin fragments; pro-C3: N-terminal propeptide of type III collagen; pro-C6: N-terminal propeptide of type VI collagen.

Correlations between baseline FVC (mL) and baseline concentrations of each biomarker are shown in supplementary table S1. Rates of decline in FVC (mL·year^−1^) in subgroups by baseline levels of biomarkers (based on the optimal cut-off) are shown in supplementary table S2. Only s-ICAM showed a significant (FDR-corrected p<0.05) association between continuous baseline level and the rate of decline in FVC over 52 weeks (estimate −122.4, 95% CI −179.2– −65.5) and time to ILD progression or death over 52 weeks (HR 2.03, 95% CI 1.33–3.11) (figures 1 and 2). None of the biomarkers showed a significant association between baseline level and time to first acute exacerbation or death, or death alone (supplementary figures S1 and S2). Associations between baseline biomarker levels and outcomes by fibrotic pattern on HRCT are shown in supplementary tables S3–S6. Baseline level of s-ICAM was significantly associated with the rate of decline in FVC over 52 weeks in subjects with a UIP-like pattern, but not in subjects with other patterns on HRCT (estimates of −162.9 (95% CI −246.9– −78.9) and −74.3 (95% CI −152.0–3.3), respectively).

### Effects of nintedanib on circulating biomarkers

**FIGURE 3:**
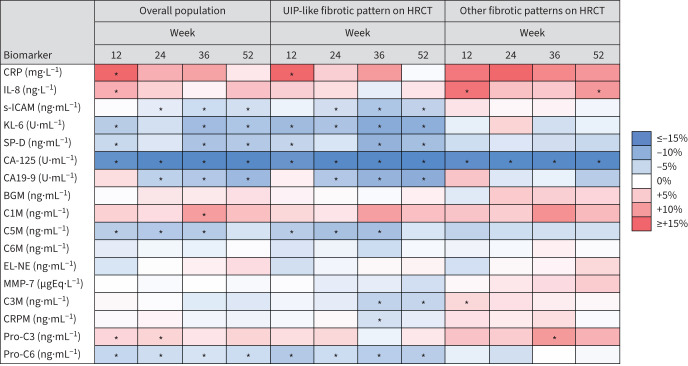
Heat map depicting the effects of nintedanib on changes in biomarkers overall and in subgroups by fibrotic pattern on high-resolution computed tomography (HRCT) in the INBUILD trial. Shading denotes the relative difference *versus* placebo in change from baseline in the biomarker. *: p≤0.05. At baseline, n=252 to n=318 for nintedanib and n=273 to n=312 for placebo in the overall population; n=156 to n=201 for nintedanib and n=174 to n=196 for placebo among subjects with usual interstitial pneumonia (UIP)-like fibrotic pattern on HRCT; n=94 to n=117 for nintedanib and n=105 to n=116 for placebo among subjects with other fibrotic patterns on HRCT. At week 52, n=177 to n=230 for nintedanib and n=182 to n=253 for placebo in the overall population; n=104 to n=142 for nintedanib and n=103 to n=152 for placebo among subjects with UIP-like fibrotic pattern on HRCT; n=65 to n=89 for nintedanib and n=76 to n=101 for placebo among subjects with other fibrotic patterns on HRCT. KL-6: Krebs von den Lungen-6; SP-D: surfactant protein D; CRP: C-reactive protein; IL-8: interleukin-8; s-ICAM: soluble intercellular adhesion molecule; BGM: biglycan degraded by MMP; C1M: collagen 1 degraded by MMP-2/9/13; C3M: collagen 3 degraded by MMP-9; C5M: collagen 5 degraded by MMP-2/9; C6M: collagen 6 degraded by MMP-2/9; EL-NE: neutrophil-specific elastin fragments; pro-C3: N-terminal propeptide of type III collagen; pro-C6: N-terminal propeptide of type VI collagen.

In the overall population, there were decreases in KL-6, SP-D, CA-125 and CA19-9 in subjects who received nintedanib *versus* placebo over 52 weeks (figure 3). The largest decrease was observed for CA-125. Increases in CRP and IL-8 were observed in subjects who received nintedanib *versus* placebo at week 12 but not at week 52. Small decreases in pro-C6 and s-ICAM were observed in subjects who received nintedanib *versus* placebo over 52 weeks, starting from week 12 and week 24, respectively. A small decrease in C5M and a small increase in pro-C3 were observed in subjects who received nintedanib *versus* placebo at weeks 12 and 24, but not at week 52. The effects of nintedanib on KL-6, SP-D, CA19-9, s-ICAM and pro-C6 appeared to be driven by the subjects with a UIP-like pattern on HRCT. The effect of nintedanib on reducing CA-125 was consistent between subgroups by HRCT pattern (figure 3).

### Mediation analyses

**FIGURE 4:**
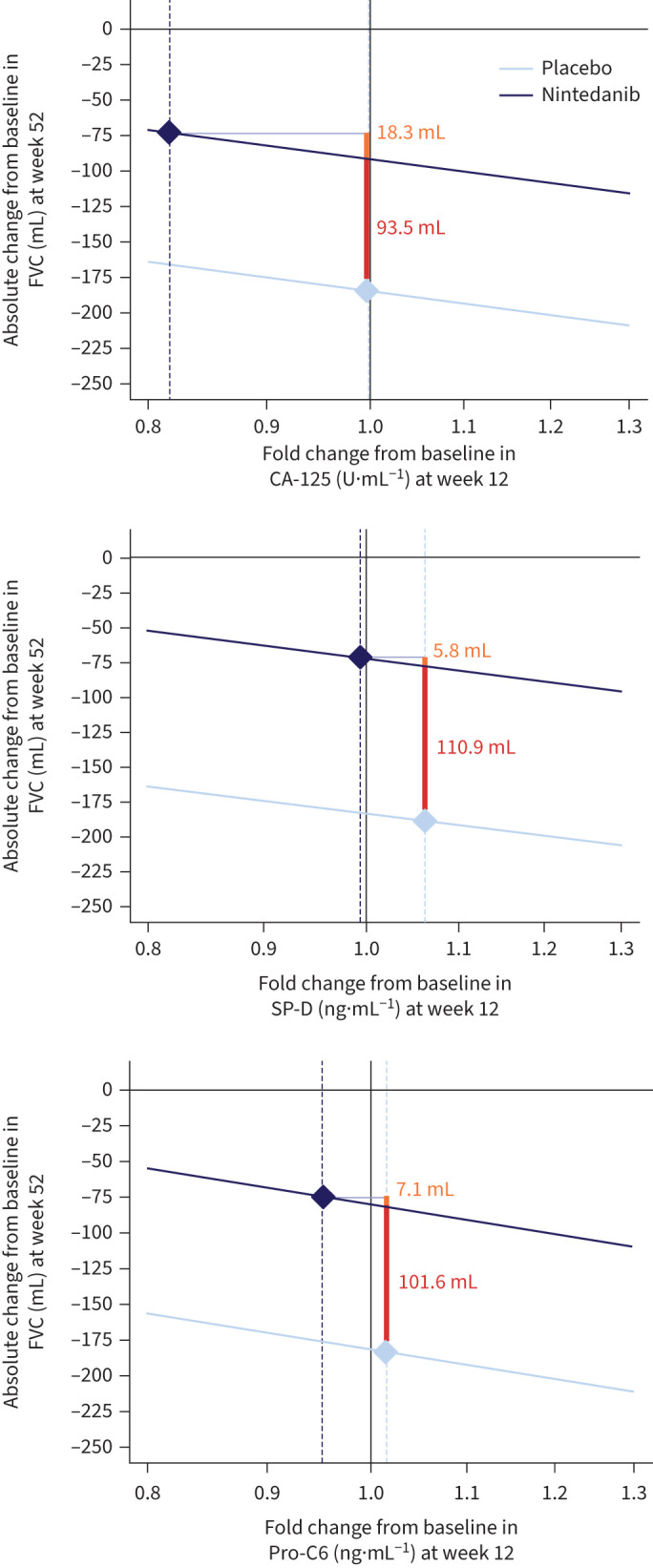
Changes in forced vital capacity (FVC) at week 52 attributable to treatment-related changes in CA-125, surfactant protein D (SP-D) and N-terminal propeptide of type VI collagen (pro-C6) at week 12. The orange values denote the indirect effect that the treatment-related change in the biomarker at week 12 has on the change in FVC (mL) at week 52. The red values denote the direct effect of nintedanib on the change in FVC (mL) not mediated by the change in the biomarker. The diamonds indicate the adjusted mean changes in the biomarker and in FVC in the nintedanib and placebo groups.

Three biomarkers met the criteria for a mediation analysis: CA-125, SP-D and pro-C6 (figure 4). In the mediation analysis of CA-125, the total treatment effect of nintedanib on change in FVC at week 52 was 111.8 mL; of this, a change of 18.3 mL (16.4%) was attributed to the treatment-related decrease in CA-125 at week 12. For SP-D, the total treatment effect of nintedanib on change in FVC at week 52 was 116.7 mL; of this, a change of 5.8 mL (5.0%) was attributed to the treatment-related decrease in SP-D at week 12. For pro-C6, the total treatment effect of nintedanib on change in FVC at week 52 was 108.6 mL; of this, a change of 7.1 mL (6.5%) was attributed to the treatment-related decrease in pro-C6 at week 12. Scatterplots depicting correlations between changes in these biomarkers at weeks 12, 24, 36 and 52 and the rate of decline in FVC over 52 weeks are shown in supplementary figures S3–S5.

## Discussion

These analyses of data from the INBUILD trial suggest that in subjects with PPF, other than ICAM-1, baseline levels of circulating biomarkers were not associated with the rate of decline in FVC over 52 weeks or other clinically relevant outcomes. Previous studies have suggested that circulating biomarkers such as CA-125, KL-6, MMP-7 and ICAM-1 are associated with ILD progression or mortality in subjects with fibrosing ILDs [14–19]; however, the sensitivity and specificity of these biomarkers appear to be limited [20–22]. To our knowledge, only one previous study of the prognostic potential of circulating biomarkers has been conducted in patients enrolled based on having PPF. In that study, among 77 subjects with PPF defined using the INBUILD trial criteria, elevated KL-6 at baseline, assessed as a continuous variable, was associated with an increased risk of death over 3 years in univariable analyses [23]. The difference in findings of our analysis compared with studies conducted in broader populations of subjects with fibrosing ILDs may reflect the fact that all the patients in the INBUILD trial were enrolled based on having progressive disease. Our current analyses of data from the INBUILD trial are consistent with those from the INMARK trial conducted in subjects with IPF and preserved lung function, in which there were no significant associations between baseline biomarker levels and disease progression over 52 weeks in analyses adjusted for multiple comparisons and the addition of biomarkers to models based on demographic and clinical factors did not improve prediction of disease progression [22].

In these analyses of data from the INBUILD trial, treatment with nintedanib reduced circulating levels of markers of epithelial injury over 52 weeks, with effects observed as early as week 12. The greatest effect of nintedanib was observed on CA-125. The effect of nintedanib on reducing CA-125 was consistent between subgroups by HRCT pattern. A mediation analysis suggested that in the overall population, approximately 16% of the effect of nintedanib on reducing decline in FVC over 52 weeks was attributed to the treatment-related change in CA-125 over 12 weeks. CA-125 is the peptide epitope of MUC16, a membrane-bound mucin that plays a key role in maintaining the epithelium [24]. MUC16 has been shown to be overexpressed in lung tissue of patients with IPF compared with healthy controls and to be localised in fibroblasts from fibrotic foci and proliferative alveolar type II cells [25]. In the PROFILE study, CA-125 was increased throughout the metaplastic epithelium in pulmonary fibrotic lesions from patients with IPF [14]. Elevated levels of CA-125 have been observed in patients with various ILDs compared with people without ILD [26–29]. Consistent with our findings, nintedanib also reduced levels of CA-125 in the INMARK trial in subjects with IPF [30] and in a trial in subjects with ILD associated with systemic sclerosis [31]. In the INMARK trial, a mediation analysis suggested that approximately 40% of the effect of nintedanib on change in FVC over 12 weeks was attributed to the change in CA-125 over 12 weeks [30]. Based on these findings, we believe that CA-125 represents a promising pharmacodynamic biomarker for nintedanib at the population level. However, large variability at the patient level limits the use of CA-125 as a biomarker for individual treatment decisions.

Strengths of our analysis include the use of data from a large, prospective, randomised placebo-controlled trial. Limitations include the need for batch correction for some of the biomarkers. The potential effect of combinations of biomarkers was not studied. Other therapies previously/currently taken by the patients in the study may have introduced confounding effects. Although nintedanib reduced circulating levels of markers of epithelial injury, it is not known whether nintedanib has a direct effect on epithelial dysfunction. The mechanisms by which nintedanib reduces levels of CA-125 are unknown.

In conclusion, data from the INBUILD trial suggest that in subjects with PPF, nintedanib may have an early effect on reducing circulating CA-125 and, to a lesser extent, other markers of epithelial dysfunction. Further studies are required to inform the clinical utility of blood biomarkers in patients with PPF.

## Data Availability

To ensure independent interpretation of clinical study results and enable authors to fulfil their roles and obligations under the ICMJE criteria, Boehringer Ingelheim grants authors access to relevant clinical study data. In adherence with the Boehringer Ingelheim Policy on Transparency and Publication of Clinical Study Data, scientific and medical researchers can request access to clinical study data, typically 1 year after the approval has been granted by major regulatory authorities or after termination of the development programme. Researchers should use https://vivli.org/ to request access to study data and visit https://www.mystudywindow.com/msw/datasharing for further information.

## Supplementary material

**Please note:** supplementary material is not edited by the Editorial Office, and is uploaded as it has been supplied by the author. Supplementary material 00158-2025.SUPPLEMENT
