# Supplementary material for: Circulating biomarkers in subjects with progressive pulmonary fibrosis: data from the INBUILD trial
Source: ERJ Open Res. 2026 Feb 23;12(1):00158-2025. doi: 10.1183/23120541.00158-2025 (PMC12926820; doi:10.1183/23120541.00158-2025)
Supplement: Supplementary file 1 [file 00158-2025.SUPPLEMENT.pdf]

Circulating biomarkers in subjects with progressive pulmonary fibrosis: data from the INBUILD trial

Online data supplement

Figure S1. Associations between baseline biomarker levels and time to first acute exacerbation or death in the INBUILD trial.

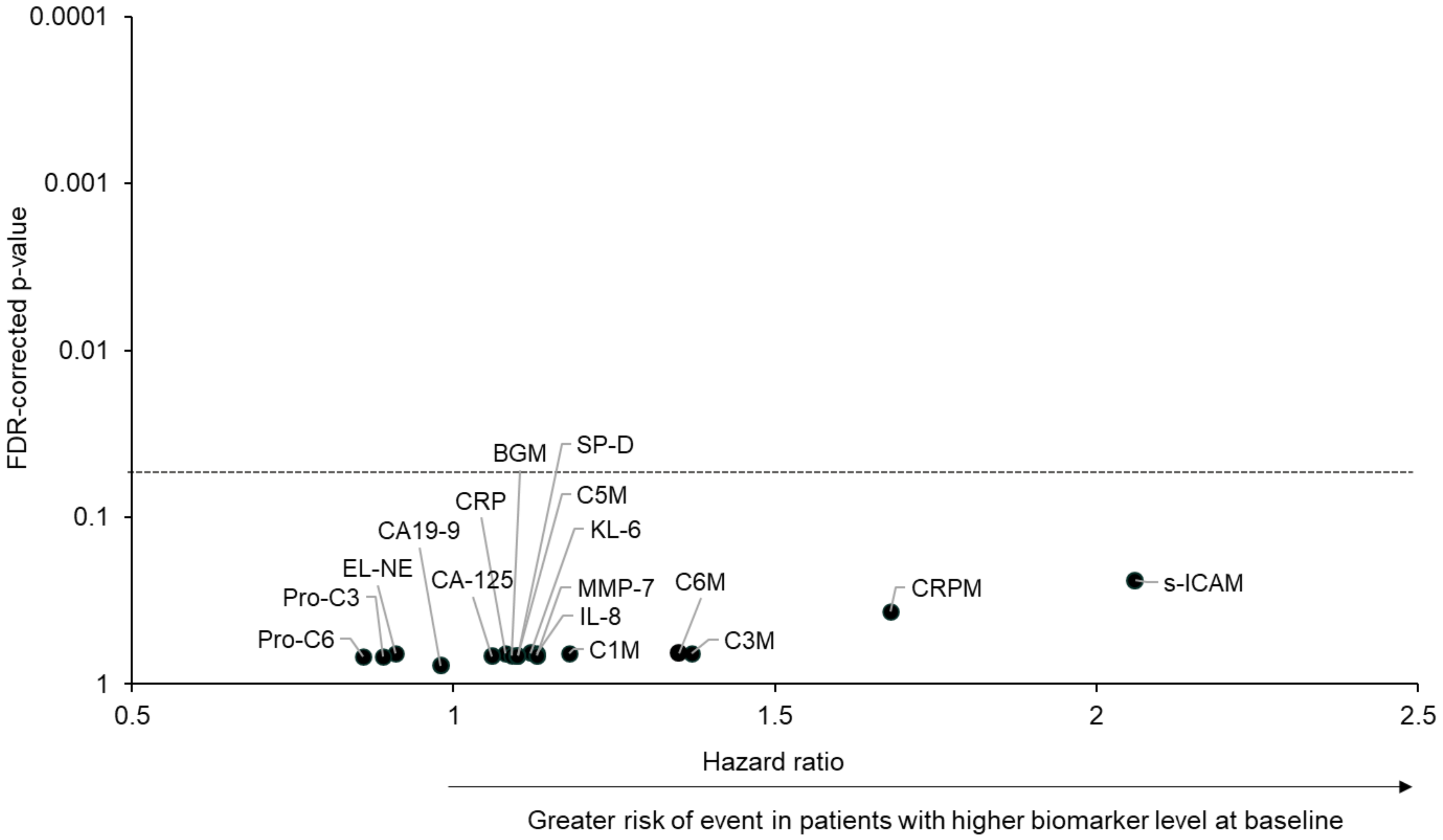

Hazard ratios represent the risk of an event associated with a difference of one in the log_2 transformed level of the biomarker at baseline.

Figure S2. Associations between baseline biomarker levels and time to death in the INBUILD trial.

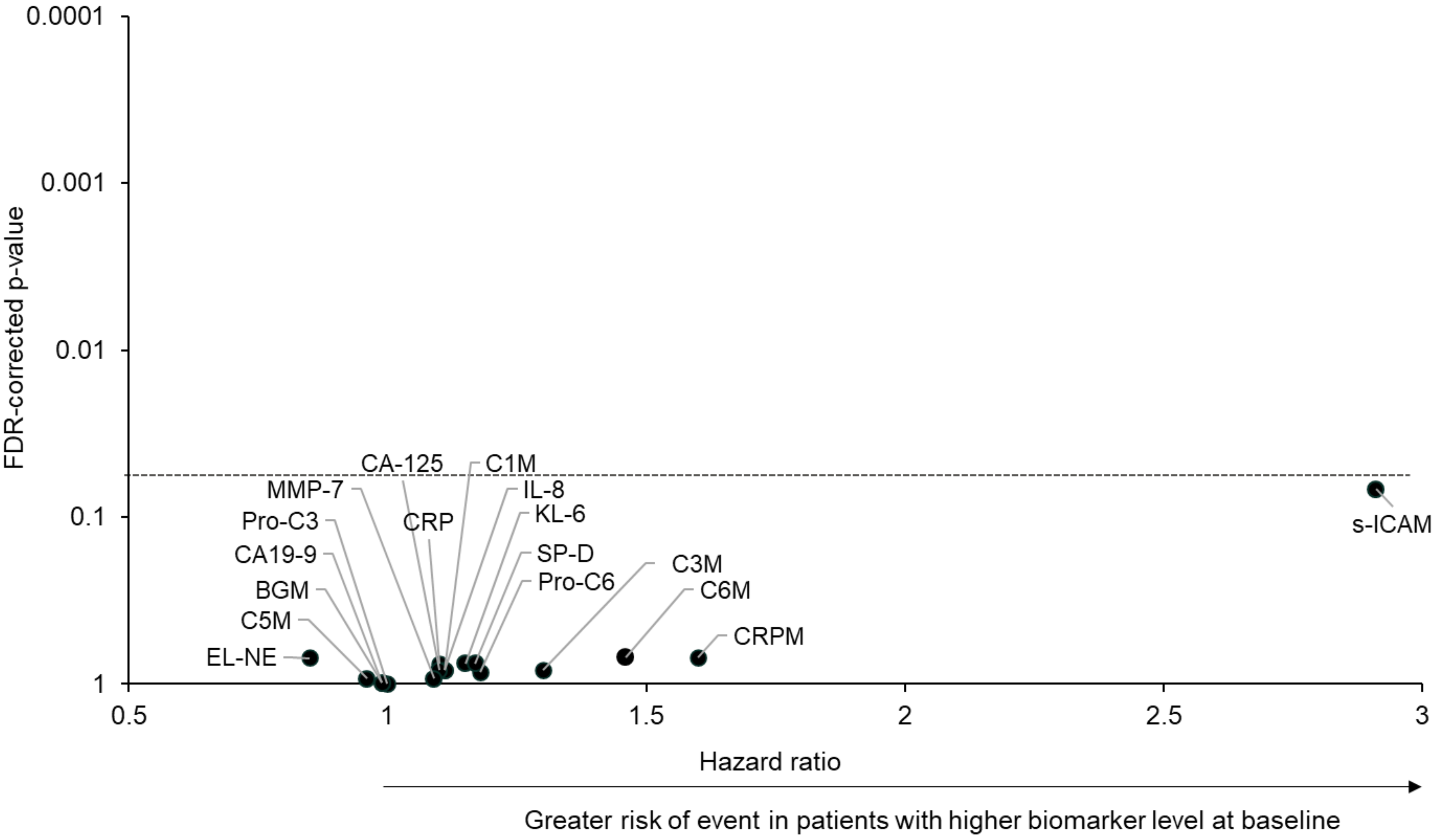

Hazard ratios represent the risk of an event associated with a difference of one in the $\log_2$ transformed level of the biomarker at baseline.

Figure S3. Correlations (rho) between change in CA-125 (U/mL) at weeks 12, 24, 36 and 52 and rate of decline in FVC (mL/year) over 52 weeks in the INBUILD trial.

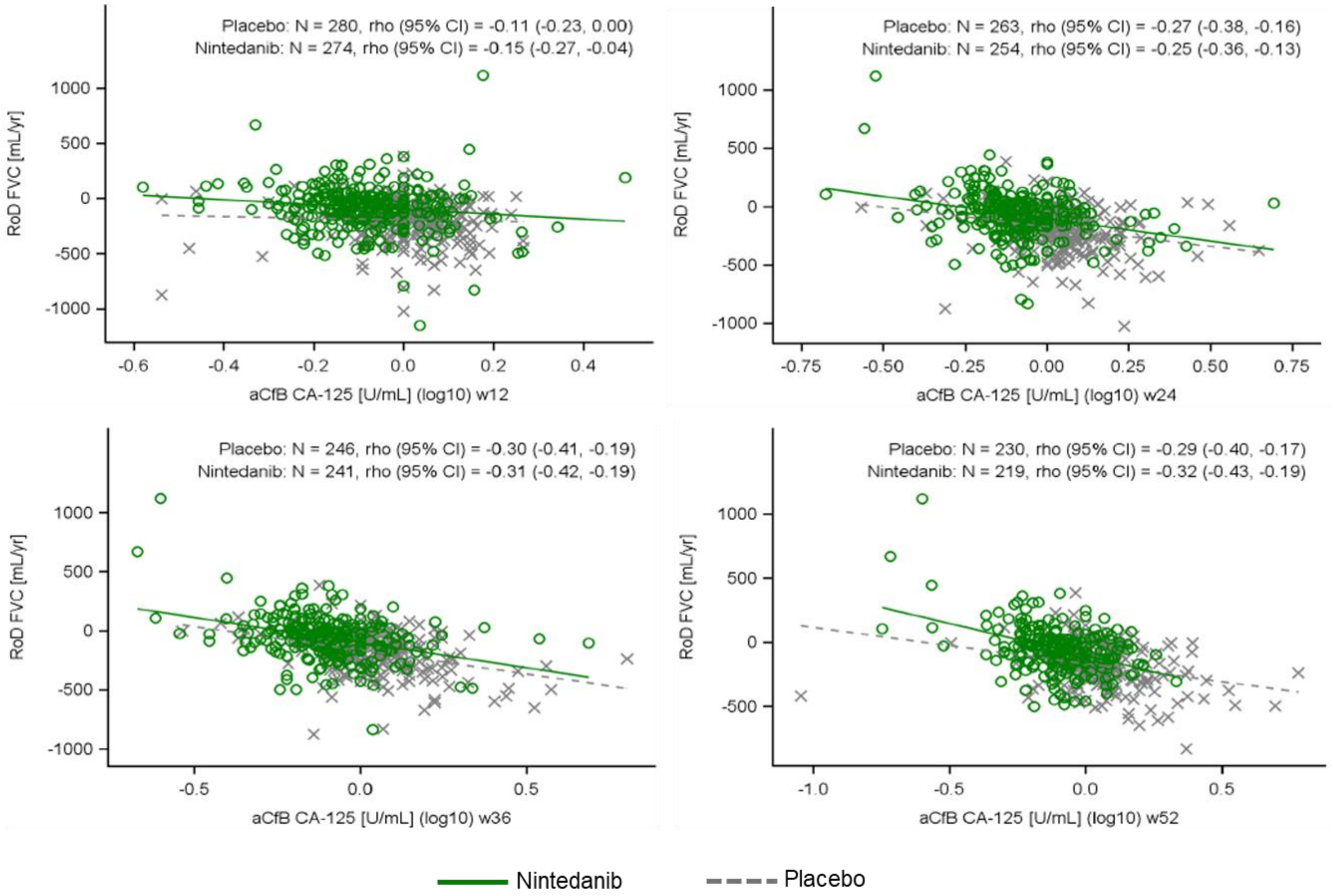

aCfB, absolute change from baseline. RoD, rate of decline

Figure S4. Correlations (rho) between change in SP-D (ng/mL) at weeks 12, 24, 36 and 52 and rate of decline in FVC (mL/year) over 52 weeks in the INBUILD trial.

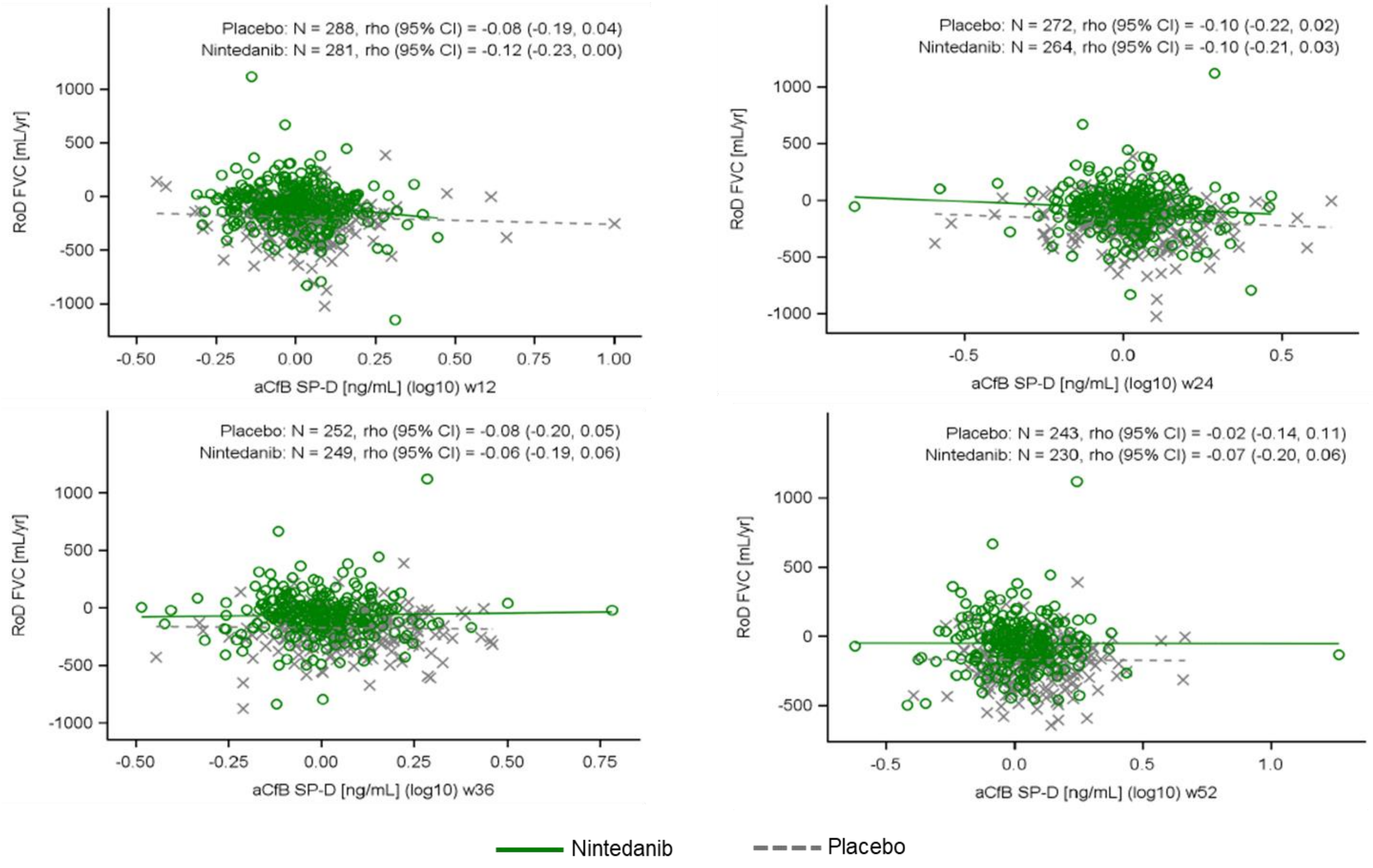

aCfB, absolute change from baseline. RoD, rate of decline

Figure S5. Correlations (rho) between change in pro-C6 (ng/mL) at weeks 12, 24, 36 and 52 and rate of decline in FVC over 52 weeks in the INBUILD trial.

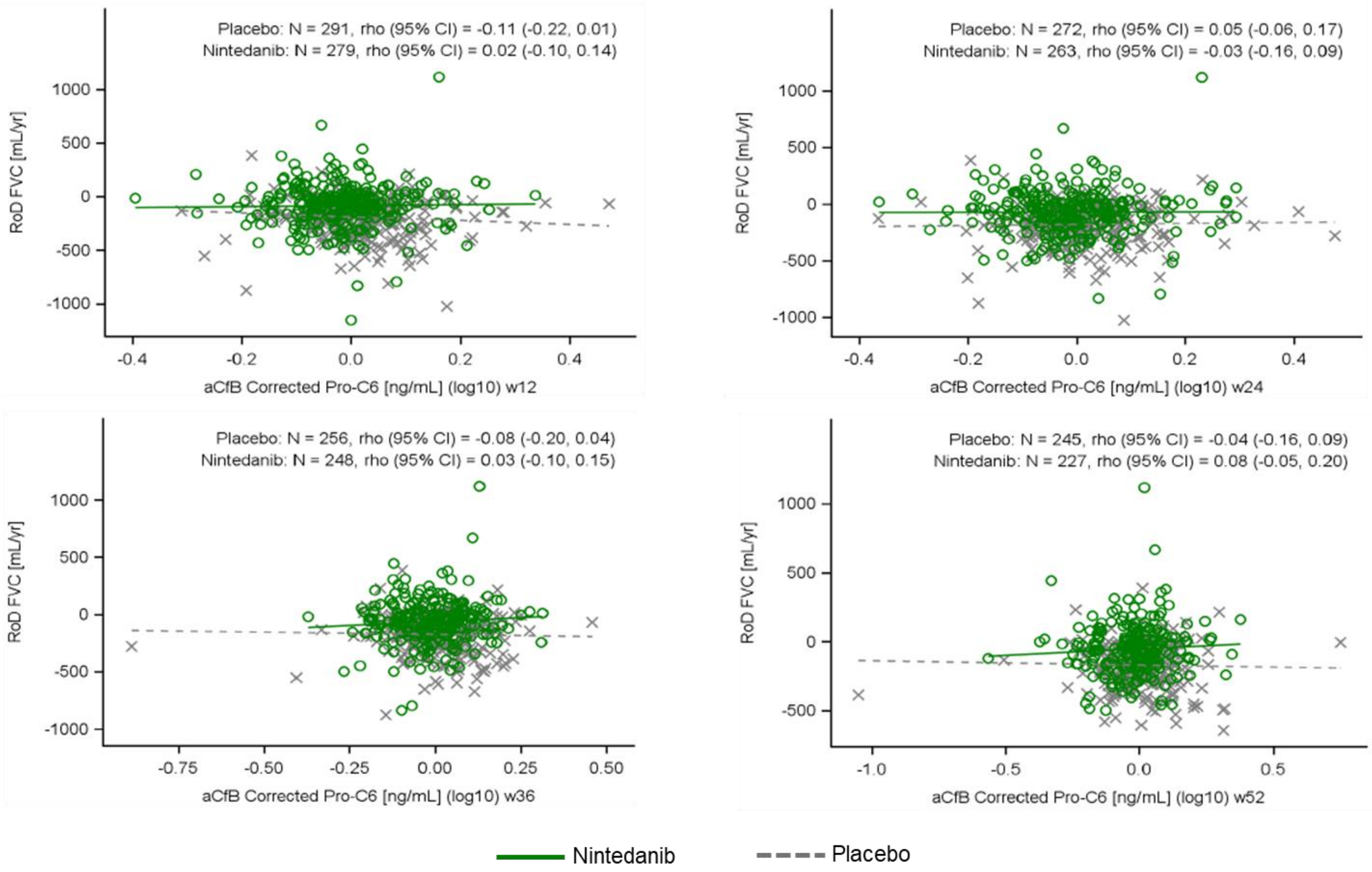

aCfB, absolute change from baseline. RoD, rate of decline

Table S1. Correlations (Spearman's rho) between FVC (mL) and raw biomarker concentrations at baseline in the INBUILD trial.

	**Correlation coefficient (95% CI)**	**P-value**
KL-6, U/mL	−0.07 (−0.15, 0.01)	0.082
SP-D, ng/mL	−0.00 (−0.08, 0.07)	0.932
CA-125, U/mL	−0.09 (−0.17, −0.01)	0.025
CA19-9, U/mL	−0.17 (−0.25, −0.09)	<0.001
CRP, mg/L	−0.02 (−0.10, 0.06)	0.637
IL-8, ng/L	−0.10 (−0.18, −0.02)	0.014
s-ICAM, ng/mL	−0.04 (−0.12, 0.03)	0.276
BGM, ng/mL	−0.07 (−0.15, 0.01)	0.076
C1M, ng/mL	−0.01 (−0.09, 0.07)	0.737
C3M, ng/mL	−0.07 (−0.14, 0.01)	0.098
C5M, ng/mL	−0.05 (−0.13, 0.03)	0.192
C6M, ng/mL	0.00 (−0.07, 0.08)	0.911
EL-NE, ng/mL	−0.04 (−0.12, 0.04)	0.370
MMP-7, ugEq/L	−0.21 (−0.29, −0.14)	<0.001
CRPM, ng/mL	0.01 (−0.07, 0.09)	0.818
Pro-C3, ng/mL	0.06 (−0.01, 0.14)	0.113
Pro-C6, ng/mL	0.01 (−0.07, 0.09)	0.756

n=571 to n=663. Correlations were based on non-log-transformed biomarker values.

Table S2. Rates of decline in FVC (mL/year) over 52 weeks in subgroups by baseline biomarker levels in the placebo group of the INBUILD trial (based on optimal thresholds for division into two subgroups).

	N	Adjusted rate (SE) of decline in FVC (mL/year)*	Adjusted difference (95% CI)*
s-ICAM, ng/mL			
≤849	242	−159.5 (15.1)	−139.9 (−203.9, −75.9)
>849	70	−299.4 (28.8)	
KL-6, U/mL			
≤1150	213	−179.7 (16.5)	−34.2 (−92.6, 24.1)
>1150	99	−214.0 (24.6)	
SP-D, ng/mL			
≤1050	240	−178.1 (15.5)	−54.6 (−119.5, 10.2)
>1050	72	−232.7 (29.0)	
CA-125, U/mL			
≤18.0	178	−176.7 (18.4)	−47.6 (−104.4, 9.2)
>18.0	128	−224.3 (22.2)	
CA19-9, U/mL			
≤13.5	108	−150.1 (23.1)	−66.4 (−124.4, −8.4)
>13.5	179	−216.5 (17.8)	
C5M, ng/mL			
≤10.2	244	−182.8 (15.6)	−28.7 (−97.3, 39.8)
>10.2	62	−211.5 (31.1)	
C6M, ng/mL			
≤22.5	92	−139.9 (24.9)	−69.5 (−128.1, −11.0)
>22.5	219	−209.4 (16.2)	
MMP-7, ugEq/L			

≤7.0	77	−139.7 (27.3)	−67.8 (−130.2, −5.4)
>7.0	235	−207.5 (15.7)	
Pro-C3, ng/mL			
≤9.6	74	−115.5 (28.1)	−95.3 (−158.6, −32.0)
>9.6	237	−210.8 (15.4)	
Pro-C6, ng/mL			
≤6.8	65	−130.0 (29.6)	−73.8 (−139.4, −8.2)
>6.8	247	−203.8 (15.3)	

*Based on a random coefficient regression with fixed effects of dichotomized biomarker by cutoff, HRCT pattern (UIP-like pattern or other fibrotic patterns), fixed continuous effects of time, baseline FVC (mL), and including dichotomized biomarker-by-time and baseline FVC-by-time interactions. The optimal threshold was determined by evaluating candidate cut-offs for each biomarker at baseline (*i.e.*, baseline level ≤*c* vs >*c*) and selecting the value that optimized the restricted maximum likelihood of the random coefficient regression. Values between the 20% quantile and the 80% quantile of each biomarker at baseline were evaluated. The cut-off which maximized the likelihood of the models was selected as the optimal threshold.

Table S3. Associations between baseline biomarker levels and rate of decline in FVC (mL/year) over 52 weeks in the placebo group of the INBUILD trial by fibrotic pattern on HRCT.

	**Estimate (95% CI) in subjects with UIP-like fibrotic pattern on HRCT**	**Estimate (95% CI) in subjects with other fibrotic patterns on HRCT**
KL-6, U/mL	−12.9 (−47.2, 21.4)	7.2 (−25.6, 40.0)
SP-D, ng/mL	−8.0 (−42.9, 26.9)	−16.6 (−44.2, 11.0)
CA-125, U/mL	−29.7 (−67.2, 7.8)	−3.5 (−40.4, 33.5)
CA19-9, U/mL	−7.3 (−26.7, 12.0)	−2.1 (−20.1, 15.8)
CRP, mg/L	9.1 (−11.6, 29.8)	−2.7 (−24.7, 19.2)
IL-8, ng/L	20.6 (−21.6, 62.8)	−45.9 (−98.1, 6.3)
s-ICAM, ng/mL	−162.9 (−246.9, −78.9)*	−74.3 (−152.0, 3.3)
BGM, ng/mL	−1.8 (−34.6, 30.9)	−17.0 (−54.8, 20.9)
C1M, ng/mL	−9.7 (−47.4, 28.0)	−17.7 (−56.6, 21.2)
C3M, ng/mL	−38.2 (−134.7, 58.4)	5.7 (−100.3, 111.7)
C5M, ng/mL	15.7 (−27.7, 59.1)	−34.0 (−72.6, 4.6)
C6M, ng/mL	−36.9 (−98.6, 24.8)	−51.9 (−133.0, 29.2)
EL-NE, ng/mL	11.8 (−17.0, 40.6)	−16.0 (−45.7, 13.7)
MMP-7, ugEq/L	−23.3 (−78.0, 31.4)	−10.5 (−62.0, 41.0)
CRPM, ng/mL	−50.4 (−129.2, 28.4)	−48.3 (−129.8, 33.2)
Pro-C3, ng/mL	1.1 (−61.3, 63.4)	−99.1 (−172.0, −26.2)
Pro-C6, ng/mL	25.1 (−56.8, 107.1)	−61.3 (−134.4, 11.8)

Estimates represent the rate of decline in FVC (mL/year) over 52 weeks associated with a difference of one in the $\log_2$ transformed level of the biomarker at baseline. *FDR-corrected $p<0.05$.

Table S4. Associations between baseline biomarker levels and time to ILD progression or death over 52 weeks in the placebo group of the INBUILD trial by fibrotic pattern on HRCT.

	**HR (95% CI) in subjects with UIP-like fibrotic pattern on HRCT**	**HR (95% CI) in subjects with other fibrotic patterns on HRCT**
KL-6, U/mL	1.14 (0.93, 1.41)	1.00 (0.74, 1.35)
SP-D, ng/mL	1.13 (0.90, 1.40)	1.37 (1.01, 1.87)
CA-125, U/mL	1.30 (1.05, 1.62)	0.95 (0.69, 1.32)
CA19-9, U/mL	1.08 (0.95, 1.23)	1.10 (0.94, 1.29)
CRP, mg/L	0.99 (0.87, 1.12)	1.01 (0.84, 1.22)
IL-8, ng/L	0.93 (0.72, 1.21)	1.84 (1.30, 2.62)*
s-ICAM, ng/mL	2.12 (1.21, 3.74)	1.83 (0.92, 3.66)
BGM, ng/mL	1.02 (0.84, 1.25)	1.02 (0.72, 1.43)
C1M, ng/mL	1.22 (0.97, 1.55)	1.06 (0.76, 1.48)
C3M, ng/mL	1.10 (0.64, 1.90)	0.93 (0.37, 2.31)
C5M, ng/mL	1.07 (0.82, 1.39)	1.01 (0.72, 1.43)
C6M, ng/mL	1.34 (0.92, 1.96)	0.84 (0.39, 1.84)
EL-NE, ng/mL	0.98 (0.82, 1.18)	1.04 (0.81, 1.33)
MMP-7, ugEq/L	1.10 (0.78, 1.55)	1.22 (0.75, 1.99)
CRPM, ng/mL	1.65 (1.02, 2.65)	1.28 (0.58, 2.81)
Pro-C3, ng/mL	0.90 (0.64, 1.29)	1.09 (0.56, 2.14)
Pro-C6, ng/mL	0.84 (0.52, 1.34)	1.25 (0.64, 2.42)

Hazard ratios represent the risk of an event associated with a difference of one in the log_2 transformed level of the biomarker at baseline. *FDR-corrected $p<0.05$.

Table S5. Associations between baseline biomarker levels and time to first acute exacerbation or death in the placebo group of the INBUILD trial by fibrotic pattern on HRCT.

	**HR (95% CI) in subjects with UIP-like fibrotic pattern on HRCT**	**HR (95% CI) in subjects with other fibrotic patterns on HRCT**
KL-6, U/mL	1.08 (0.82, 1.41)	1.16 (0.75, 1.81)
SP-D, ng/mL	1.05 (0.79, 1.40)	1.19 (0.75, 1.88)
CA-125, U/mL	1.11 (0.83, 1.48)	0.89 (0.53, 1.49)
CA19-9, U/mL	1.01 (0.86, 1.18)	0.95 (0.78, 1.15)
CRP, mg/L	1.06 (0.91, 1.24)	1.22 (0.88, 1.69)
IL-8, ng/L	1.07 (0.83, 1.39)	1.38 (0.81, 2.35)
s-ICAM, ng/mL	2.70 (1.28, 5.69)	1.33 (0.51, 3.41)
BGM, ng/mL	0.96 (0.75, 1.22)	2.11 (1.09, 4.07)
C1M, ng/mL	0.97 (0.71, 1.33)	2.14 (1.21, 3.81)
C3M, ng/mL	1.26 (0.61, 2.58)	2.00 (0.48, 8.38)
C5M, ng/mL	0.99 (0.73, 1.34)	1.91 (0.79, 4.59)
C6M, ng/mL	1.30 (0.80, 2.11)	1.71 (0.60, 4.89)
EL-NE, ng/mL	0.78 (0.62, 0.98)	1.54 (0.99, 2.39)
MMP-7, ugEq/L	0.94 (0.62, 1.43)	1.70 (0.87, 3.31)
CRPM, ng/mL	1.73 (0.95, 3.15)	1.75 (0.63, 4.89)
Pro-C3, ng/mL	0.75 (0.46, 1.22)	1.92 (0.70, 5.25)
Pro-C6, ng/mL	0.69 (0.37, 1.30)	1.68 (0.63, 4.48)

Hazard ratios represent the risk of an event associated with a difference of one in the log_2 transformed level of the biomarker at baseline. All FDR-corrected p>0.05.

Table S5. Associations between baseline biomarker levels and time to death in the placebo group of the INBUILD trial by fibrotic pattern on HRCT.

	**HR (95% CI) in subjects with UIP-like fibrotic pattern on HRCT**	**HR (95% CI) in subjects with other fibrotic patterns on HRCT**
KL-6, U/mL	1.10 (0.81, 1.49)	1.07 (0.52, 2.18)
SP-D, ng/mL	1.14 (0.82, 1.58)	0.97 (0.46, 2.06)
CA-125, U/mL	1.15 (0.83, 1.60)	0.80 (0.34, 1.89)
CA19-9, U/mL	1.02 (0.86, 1.22)	0.87 (0.66, 1.13)
CRP, mg/L	1.06 (0.88, 1.27)	1.49 (0.84, 2.64)
IL-8, ng/L	1.03 (0.77, 1.38)	1.95 (0.86, 4.43)
s-ICAM, ng/mL	3.71 (1.55, 8.87)	0.89 (0.19, 4.11)
BGM, ng/mL	0.89 (0.68, 1.17)	2.07 (0.71, 6.06)
C1M, ng/mL	0.84 (0.58, 1.20)	12.60 (2.49, 63.81)*
C3M, ng/mL	1.26 (0.55, 2.86)	2.11 (0.20, 22.20)
C5M, ng/mL	0.92 (0.67, 1.28)	1.00 (0.40, 2.48)
C6M, ng/mL	1.26 (0.72, 2.18)	10.00 (1.57, 63.73)
EL-NE, ng/mL	0.76 (0.58, 0.99)	1.48 (0.77, 2.84)
MMP-7, ugEq/L	0.94 (0.58, 1.53)	2.47 (0.82, 7.46)
CRPM, ng/mL	1.51 (0.75, 3.02)	4.87 (0.75, 31.57)
Pro-C3, ng/mL	0.91 (0.53, 1.56)	1.94 (0.44, 8.61)
Pro-C6, ng/mL	0.97 (0.48, 1.98)	3.75 (0.59, 23.75)

Hazard ratios represent the risk of an event associated with a difference of one in the log_2 transformed level of the biomarker at baseline. *FDR-corrected $p<0.05$.
